# Pain Assessment in Patients during Hemodialysis Treatment: Quality Improvement Project

**DOI:** 10.3390/nursrep14020103

**Published:** 2024-05-30

**Authors:** Rita Rodrigues, Cristina Costeira

**Affiliations:** 1 School of Health Sciences of Polytechnic of Leiria, Campus 2, Morro do Lena, Alto do Vieiro, Apartado 4137, 2411-901 Leiria, Portugal; rita.rodrigues@chleiria.min-saude.pt; 2 Local Unit of Leiria, R. de Santo André, 2410-197 Leiria, Portugal; 3 Centre for Innovative Care and Health Technology (ciTechCare), Campus 5, Polytechnic of Leiria, Rua das Olhalvas, 2414-016 Leiria, Portugal; 4 Health Sciences Research Unit: Nursing (UICISA: E), Nursing School of Coimbra (ESEnfC), 3004-011 Coimbra, Portugal

**Keywords:** nursing care, pain, chronic kidney disease, hemodialysis

## Abstract

Pain is a prevalent symptom in patients with chronic kidney disease, related to disease progression, comorbidities, and required immobility during dialysis treatment. Nurses must perform detailed pain assessments to manage pain effectively during hemodialysis treatment. This quality improvement project, reported using SQUIRE 2.0, aims to describe pain characteristics in chronic kidney disease patients in a hemodialysis unit clinic in Portugal, implement strategies to improve the pain assessment process in patients with chronic kidney disease during hemodialysis treatment, and assess nurses’ satisfaction with the implemented strategies. The study was conducted in a Portuguese hemodialysis clinic, with patients and nurses, in three phases: diagnostic study, protocol implementation, and a descriptive study to assess nurse satisfaction. Seventy-five patients (mean age 71 ± 12.6 years) participated, with 64% reporting moderate chronic pain daily and 48% during hemodialysis treatment. Thirteen nurses considered the pain assessment protocol important, rating their satisfaction at 7.92 ± 1.32 (0–10). Standardizing practices through protocol implementation is likely to improve care and increase nurse satisfaction.

## 1. Introduction

The World Health Organization (WHO) considers chronic diseases to be one of the greatest threats to the population in the 21st century, capable of affecting anyone, anywhere in the world. In 2000, chronic diseases were responsible for 61% of the causes of death worldwide, and in 2019 this rate increased to 74% [1].

In Portugal, chronic kidney disease (CKD) is one of the most prevalent and high-incidence chronic diseases, leading to significant treatment costs [2]. The National Kidney Foundation states that CKD is a progressive condition with five stages. In the first stage, there are no clinically or laboratory-significant alterations because the healthy nephrons compensate for the loss of function in others. From the second to the fourth stage, there is a gradual decline in kidney function. The fifth and final stage represents renal failure, necessitating the initiation of renal replacement therapy, such as hemodialysis (HD) or kidney transplantation [3].

The preferred renal replacement treatment is HD, which partially replaces the functions of the kidney, particularly in blood filtration and fluid elimination. It is estimated that 90% of patients with CKD undergo HD to sustain life [4]. In Portugal, in 2022, there were 12,878 individuals receiving HD treatment [5].

Hemodialysis (HD) treatments increase the average life expectancy of CKD patients, but they also have various adverse effects such as pain, cramps, nausea, vomiting, and hypotension, among other complications that negatively impact the patient’s quality of life (QoL) [6]. Complications affect the physical level, as well as the psychosocial and spiritual levels, demanding person-centered care from health professionals. Only by understanding the full complexity of CKD can effective treatments be provided, thereby contributing to patient QoL [7]. Pain is one of the most common dialysis complications, accounting for approximately 40% of patient complaints [8], causing limitations and disabilities that affect QoL and patient rehabilitation. Despite this well-known reality, pain management remains neglected and ineffective [9].

### 1.1. Pain Context

Pain is defined as “an unpleasant sensory and emotional experience associated with, or resembling that associated with, actual or potential tissue damage” [10] (p. 1977). It can be classified based on different criteria, including duration (i.e., acute or chronic), type (i.e., nociceptive or neuropathic), and pathophysiological mechanism [10].

Acute pain is often a symptom of illness or injury. It is short-lived, and typically resolves within a few days or weeks, or when the triggering factor disappears. Chronic pain, on the other hand, is defined as pain that persists for more than three months. It is considered a disease that causes weakness and profound depression, affecting the individual’s daily activities and diminishing their quality of life [11].

Currently, the approach to chronic pain considers not only physical symptoms but also the interaction of various biological and psychosocial factors [12]. Chronic pain is often associated with high levels of anxiety and depression [13].

The perception of a painful stimulus associated with chronic pain is followed by cognitive processing and interpretation, leading to various behaviors and responses by those who experience it. This cognitive processing is influenced by personal beliefs, values, and the environment [14]. Approaching pain from a biopsychosocial model perspective reveals that the early detection of psychosocial factors interfering with pain can alter therapeutic strategies and expedite effective pain treatment.

The experience of pain is considered a public health problem. It is estimated that 30% of the world’s population suffers from pain, placing enormous pressure on health services [15,16]. Consequently, issues related to efficient pain management have become prominent in recent years, leading to the development of various theoretical models.

### 1.2. Pain in Patients with Chronic Kidney Disease

Pain in patients with CKD can have several causes, including disease progression (e.g., renal bone disease), procedures during HD treatments, and the presence of underlying diseases or comorbidities (e.g., ischemic peripheral arterial disease or diabetic neuropathy). Pain is more commonly reported in the upper and lower limbs, chest, and abdomen [17].

CKD patients describe their pain as a “fine, perceptible twinge” that is bothersome and leads to exhaustion. Regarding its intensity, the pain is predominantly classified using the Visual Analogue Scale (VAS) as moderate (67.8%), followed by severe pain (27.8%) [18].

### 1.3. Pain Management and Treatment

Due to the high prevalence of chronic pain in the general population, a paradigm shift in the approach to pain is essential. This involves a multi-professional and multimodal approach to pain management, focusing not only on pharmacological treatments but also on complementing them with non-pharmacological treatments [19].

In Portugal, the Directorate-General of Health (2003) mandates that pain should be treated as the fifth vital sign, requiring systematic assessment and regular clinical documentation to improve patient QoL and prioritize nursing care for patients suffering from pain. Adequate pain assessment is essential for developing appropriate management and treatment strategies [20].

The Portuguese Nurses Regulator has published guidelines outlining the responsibilities of nurses in delivering efficient pain care. Key points include implementing evidence-based strategies for effective pain control, approaching pain as a multidimensional symptom and a unique, dynamic experience, ensuring continuous professional training in pain management, prioritizing pain prevention, and advocating for policy improvements and adequate resource allocation to support effective pain control [21].

To achieve efficient pain management, appropriate pain assessment tools should be used. These include unidimensional and multidimensional pain intensity assessment instruments [11]. Unidimensional tools assess pain using a numerical or qualitative value, such as the Visual Analogue Scale (VAS), the Numeric Pain Rating Scale (NPRS), and the Faces Pain Rating Scale, among others. Multidimensional scales evaluate pain across different dimensions, including sensory, emotional–affective, and cognitive–evaluative aspects, as well as current illnesses and associated symptoms, such as the McGill Pain Questionnaire and the Brief Pain Inventory (BPI) [21]. Proper pain assessment should utilize a validated instrument that allows for an accurate evaluation of pain intensity, leading to the appropriate pain relief interventions [22].

Currently, the most widely used strategies for pain relief involve a pharmacological approach [8]. However, non-pharmacological strategies should also be applied. For example, muscle relaxation techniques are considered effective in reducing pain and improving the patient’s QoL [23]. Other effective non-pharmacological strategies include breathing techniques, acupressure, and music therapy, which have been shown to reduce pain perception in these patients [24]. The application of hot compresses during HD treatment has also been effective in reducing and relieving pain [25]. The complementarity of pharmacological and non-pharmacological approaches is beneficial for pain management, but these strategies should be prescribed and adjusted to each person’s characteristics and needs [26].

Ineffectively managed pain has consequences not only for the patients themselves but also for society. The indirect costs are significant, particularly due to loss of productivity through absenteeism and presenteeism, as well as the allocation of compensation and allowances [27].

Given the repercussions of pain, this study aimed to (i) describe pain characteristics in CKD patients in one HD unit clinic in central Portugal between November and December 2022, (ii) implement strategies to improve the pain assessment process in patients with CKD during HD treatment, and (iii) assess nurses’ satisfaction with the implemented strategies.

## 2. Materials and Methods

### 2.1. Study Design

**Figure 1:**
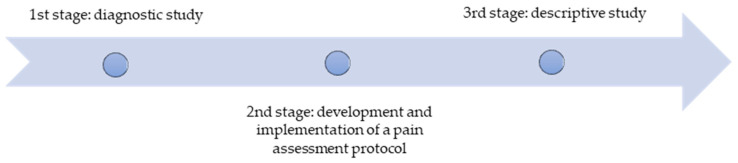
Overview of the study phases.

The quality improvement project was conducted in three main stages (Figure 1). The first stage consisted of a diagnostic study to identify key areas for improving nursing care for patients with CKD. This involved a survey applied to patients experiencing pain during HD treatment, with questions characterizing their pain. Clinical records were also used to collect some data. The survey was conducted by the nurses providing care. Each session lasted 30 min. This stage was developed in two months (November and December 2022).

The second stage, which lasted approximately four months (January to April 2023), involved developing and implementing improvement strategies based on recent scientific evidence. Those strategies were planned with the purpose of improving the data obtained in stage one. The planned strategy included a training program and the creation of a pain assessment protocol to be used during HD treatments.

The training program consisted of a 60 min face-to-face session led by the principal researcher and a nurse specialist in pain management (a member of the nursing team). All participants were invited to join in small groups to practice and discuss pain assessment instruments and pain management strategies. In the last ten minutes of the session, feedback on difficulties and suggestions for improving the protocol implementation were collected. Following these training sessions, the protocol was revised based on team suggestions and then implemented.

The training sessions were repeated twice in April to include all members of the nursing team. Eight nurses attended the first session, and twelve attended the second.

The protocol was designed in two phases. In the first phase, the principal researcher used the most recent evidence to draft a preliminary protocol. In the second phase, the researcher and a pain management specialist, who participated in the training program, revised the protocol together, leading to the implementation of the final version.

The pain assessment protocol helps nurses to standardize care and make better decisions. It specifies actions to be taken, particularly in the first hour of treatment. The steps are as follows:

(i) Assess the patient’s ability to evaluate their pain.
(ii) If the patient can assess their pain, instruments such as the Numerical Rating Pain Scale (NRPS), Visual Analogue Scale (VAS), or qualitative self-report scales should be used, as suggested by evidence, respecting the patient’s preference.
(iii) If the patient is unable to verbally assess their pain, use the Pain Assessment in Advanced Dementia (PAINAD) and Behavior Pain Scale (BPS) instruments.
(iv) If the patient reports feeling pain, the nurse should implement non-pharmacological pain relief strategies (e.g., positioning or optimizing the environment such as light, noise, and temperature control) and begin administering painkillers based on WHO recommendations and medical prescriptions. If necessary, contact the physician to readjust pharmacological prescriptions.
(v) Document nursing records, including pain characteristics (i.e., type, intensity, location, irradiation, and previous effective management strategies) and interventions performed.
(vi) Reassess pain frequently (ten minutes after administering painkillers).
(vii) During these procedures, the nurse should monitor the patient’s condition, promote comfort and well-being, and provide reassurance to the patient.

In the third stage, a self-completion online survey was sent to the nursing team to assess their satisfaction with the implemented strategies (pain assessment protocol and training session). This was completed through a descriptive study, during May and June 2023.

### 2.2. Setting of Study

The quality improvement project was conducted in an HD clinic in Portugal from November 2022 to June 2023. The clinic is part of a private management group that operates several clinics in Portugal and worldwide. Its mission is to improve the quality of life of CKD patients. The multidisciplinary team consists of nurses, physicians, operational assistants, a pharmacist, a nutritionist, a social worker, and administrative staff. It is recommended that each patient attend HD treatments three times a week, with each session lasting four hours. Exceptions are made based on the specific characteristics of each patient, which may require an increase in the number of weekly sessions and adjustments to treatment duration.

The Revised Standards for Quality Improvement Reporting Excellence Guide (SQUIRE 2.0) (Appendix A) was followed to report this project.

### 2.3. Population and Sample

In the diagnostic study (first stage), 75 out of 94 CKD patients participated in the study, representing 79.8% of the patients undergoing a regular HD program between November and December 2022. They were recruited through the care nurse by a direct invitation to participate in the study. The principal researcher (R.R.) was not involved in the data collection, to minimize bias. The clinical records of the participating patients were consulted to collect some data after their consent.

In the second stage, the strategies were implemented by 20 nurses who provide care in the HD unit.

In the descriptive study (third stage), 13 nurses chose to participate, representing 65% of the nursing staff.

#### Inclusion and Exclusion Criteria

To recruit the sample for the first stage of the diagnostic study, the following inclusion criteria were considered: patients with stage 5 CKD, on a regular HD program for one year or more, and aged 18 or over.

Regarding exclusion criteria, all patients who were unable to answer the questions were excluded (*n* = 19). All patients who met the inclusion criteria agreed to participate in the study.

The third stage of the project included all the nurses in the service, except for the trainer nurse and manager nurse, resulting in 20 eligible nurses. All participated in the training program, but only thirteen answered the online questionnaire sent to their professional email addresses during May–June 2022. The email explained the study and requested their consent.

### 2.4. Data Collection Instruments

Data were collected using two instruments developed by the researcher, both in Portuguese: one for patients (first stage) and the other for nurses (third stage).

The instrument for patients consisted of 26 closed-ended questions divided into four parts: (a) sociodemographic characterization (5 questions); (b) clinical characterization (5 questions); (c) daily pain characterization (8 questions); and (d) pain characterization during HD treatment (8 questions). The response options are represented in the tables below. Some data were collected from clinical records (Table 1 and Table 2), while the other information was gathered from patients’ responses (Table 3 and Table 4). This was a straightforward paper survey, completed by nurses using clinical records and patients’ responses during their HD treatment. It took an average of 10 min to complete.

For content validation, a pre-test was conducted with five nurses from another HD clinic under the same management, and with five patients who agreed to participate. Based on their feedback, some questions were revised. For example, possible responses about pain characterization were inserted, as both patients and nurses noted the difficulty patients had in describing their pain with adjectives. Adjectives more commonly used in clinical practice were suggested to help patients with identification.

The online survey sent to nurses (third stage) consisted of 18 closed-ended questions and two open-ended questions regarding suggestions for improving the protocol and the approach to pain during HD treatment. It was divided into two parts: (a) sociodemographic and professional characterization (6 questions); and (b) nurses’ satisfaction with the protocol assessment and its implementation (12 questions). This self-completion online survey was sent by email after the implementation phase and took an average of 5 min to complete.

This questionnaire was emailed to the same five nurses who participated in the content validation of the first questionnaire, following the same process. No changes were suggested for this instrument.

### 2.5. Data Analysis

The data obtained were statistically processed using the Statistical Program for Social Sciences^®^ (SPSS^®^) version 28.0. Descriptive statistics were used for data analysis, including absolute frequencies (n) and relative frequencies (%), measures of central tendency or location (mean, M), and measures of dispersion or variability (standard deviation, s).

For the open-ended questions in the online survey (third stage) regarding suggestions for improving the approach to pain during HD treatment and suggestions for protocol improvement, the answers were categorized through content analysis. For the first question, two categories were created: training and non-pharmacological pain management. For the second question, one category was created: nursing records.

### 2.6. Ethical Procedures

A favorable opinion was obtained from the clinic’s Ethics Committee, with code no. 7/2022. Informed consent was obtained from all participants in both the first and third stages.

The ethical principles outlined in the Declaration of Helsinki were ensured and respected throughout the study. It was guaranteed that the surveys did not identify participants and respected their right to self-determination, allowing them to withdraw at any stage of the research.

## 3. Results

### 3.1. Sociodemographic and Clinical Characterization of HD Treatments

In the first stage of the study, 75 CKD patients undergoing HD treatments and experiencing pain were predominantly men (64%), with an average age of 71.48 ± 12.70 years, ranging from 35 to 93 years old (Table 1). Regarding their residence, 81.3% lived in rural areas, and 77.3% did not have a degree.

The patients had been undergoing HD treatments for an average of 4.72 ± 3.91 years, with 98.7% receiving HD treatments three times per week. Regarding vascular access, 86.7% of the patients had an arteriovenous fistula (AVF), mostly located in the left upper limb (77.3%).

**Table 1:** Sociodemographic Characterization of Patients.

		M	SD	Max	Min
Age * (years)		71.48	12.70	93	35
		*n*	%		
Gender *	Male	48	64.00		
	Female	27	36.00		
Residence *	Countryside	61	81.30		
	Urban	14	18.70		
Qualification (by Portuguese classification) *	Illiterate (not able to read or write)	11	14.70		
	Elementary School (1st–4rd year)	45	60.00		
	1st Basic Education (5th–7th year)	7	9.30		
	2nd Basic Education (7th–9th year)	6	8.00		
	Secondary Education (10th–12nd year)	3	4.00		
	Higher Education	3	4.00		

M—Mean; SD—Standard Deviation; *n*—Sample size; %—percentage; Max—Maximum; Min—Minimum; * data collected obtained through clinical records.

**Table 2:** Summary of Clinical Characterization of HD Treatments.

		M	SD	Max	Min
Average time since starting dialysis treatment * (years)		4.72	3.91	18	1
		*n*	%		
Number of treatments *per* week *	3 times a week	74	98.70		
	4 times a week	1	1.30		
Type of vascular access *	Arteriovenous fistulae (AVF)	65	86.70		
	Polytetrafluoroethylene -PTFE	6	8.00		
	Central venous catheter (CVC)	4	5.30		
Vascular access localization *	Left upper limb	58	77.30		
	Right upper limb	13	17.30		
	Central venous catheter (CVC)	4	5.30		

M—mean; SD—standard deviation; *n*—sample size; %—percentage; Max—maximum; Min—minimum; * data obtained from clinical records.

**Table 3:** Characteristics of Patients’ Daily Pain.

		M	SD	Max	Min
Average Time with Pain (years)		3.64	2.7	10	1
Questions:		*n*	%		
Do you usually have pain?	Yes	48	64.00		
	No	27	36.00		
How is your pain duration?	Intermittent	46	61.30		
	Continued	2	2.70		
Where do you feel your pain?	Lower limbs	15	20.00		
	Lower back	15	20.00		
	Bones and muscles	14	18.70		
	Head	3	4.00		
	Other	1	1.30		
Does the pain irradiate?	No	38	50.70		
	Yes **	10	13.30		
** Where is the place where it radiates?	Lower limbs	7	9.30		
	Lower backlight shoulder	2	2.70		
	Right shoulder	1	1.30		
How could you define your pain?	“Crushing”	24	32.00		
	“Cramp”	14	18.70		
	“Stabbing”	6	8.00		
	“Tingling”	2	2.70		
	“Burning”	2	2.70		
What is the pain assessment tool used?	Qualitative Pain Scale	35	46.70		
	Numerical Rating Pain Scale (NRPS) [0–10]	13	17.30		
Do you usually do pain control medication?	Yes	27	36.00		
	No	21	28.00		
What medication do you usually take?	Paracetamol	13	17.30		
	Magnesium-metamizole	3	4.00		
	Tramadol	3	4.00		
	Pregabalin	2	2.70		
	Dot Know or remember the name	6	8.00		
Does your pain limit your Activities of Daily Living (ADLs)?	Yes **	24	32.00		
	No	24	32.00		
** What are the mostly affected ADLs?	Mobility	22	29.30		
	Sleep	2	2.70		
Are you followed by a Chronic Pain Unit team?	No	34	45.30		
	Yes	2	2.70		

M—mean; SD—standard deviation; n—sample size; %—percentage; Max—maximum; Min—minimum; ** lead to the next question.

**Table 4:** Characterization of Pain During a Hemodialysis Treatment.

		*n*	%
Do you usually have pain during your HD treatment?	No	39	52.00
	Yes	36	48.00
Where you usually have more pain?	Lower limbs	14	18.70
	Lower back	14	18.70
	Upper limbs	3	4.00
	Headaches	3	4.00
	Bones/muscles	1	1.30
	Other	1	1.30
How can you define your pain?	“Crushing”	15	20.00
	“Cramp”	9	12.00
	“Stabbing”	8	10.70
	“Tingling”	2	2.70
	“Burning”	2	2.70
Does your pain get worse during a HD treatment?	Yes **	28	37.30
	No	8	10.70
** What is your pain relief strategy?	Positioning	24	32.00
	Medication	11	14.70
	Other	1	1.30
Do you usually take painkillers during HD treatment?	No	25	33.30
	Yes **	11	14.70
** What painkiller(s) you usually do in HD treatment?	Paracetamol	8	10.70
	Magnesium-metamizole	3	4.00

M—mean; %—percentage. ** lead to the next question.

### 3.2. Characterization of Pain in Patients with CKD

Patients undergoing HD treatment reported experiencing daily pain (64%) (Table 3). However, 23 of these patients were unable to specify the chronological onset of their pain. They characterized their pain as intermittent (61.3%), predominantly in the lower limbs (20%) and lower back (20%), followed by bone and muscle pain (18.7%). The most common pain characteristics mentioned by the patients were grinding (32.0%) and stabbing (18.7%).

Regarding the radiation of pain, 50.7% reported that their pain did not radiate to any other area of the body. Among those whose pain did radiate, 9.3% mentioned the lower limbs. 

The intensity of the pain using NRPS was averaged for 13 patients, resulting 5.46 (0–10). Although 35 patients preferred to use the qualitative pain scale to assess their pain: 5 reported mild pain, 23 moderate pain, and 7 severe pain. The most used pain intensity assessment scale was the Qualitative Scale, used by 46.70% of patients, while only 17.30% used the Numerical Scale (Table 3).

When asked if they usually self-administered pain medication, 36% said “yes”. Of those, 17.3% reported using paracetamol (painkiller), 4% used magnesium-metamizole (non-acid, non-opioid), and another 4% used tramadol (weak opioid) (Table 3).

Regarding the question of whether pain limited activities of daily life (ADLs), the responses were evenly split, with 32% of patients answering “yes” and 32% answering “no.” Among those who answered “yes,” the majority (29.3%) identified mobility as the ADL that caused them the most limitations.

When asked if they were followed by a pain management team, only 2.7% (*n* = 2) said “yes.” (Table 3).

Patients undergoing HD treatment reported experiencing pain (48%). The most identified locations were the lower limbs and lower back (18.7%) (Table 4). The most frequently mentioned pain characteristic was defined as “crushing” (20%), followed by “cramp” (12%) and “stabbing” (10.7%) (Table 4).

Notably, 37.3% of the patients reported that their pain worsened during HD treatment (Table 4).

When asked about factors that aggravated their pain, eleven patients indicated that the immobility inherent to the treatment exacerbated their pain. Regarding pain relief strategies, 32% of the patients mentioned comfort positioning, while 14.7% identified the use of analgesic medication (Table 4).

The most regularly used therapies for pain relief were pharmacological measures, with paracetamol being used by 10.7% and magnesium-metamizole by 4% of the patients (Table 4).

### 3.3. Nurses’ Characterization

In the final phase of the study (third stage), after implementing the planned strategies for assessing patient pain during HD treatments, nurses were asked about their satisfaction with the protocol and training session developed.

Table 5 shows the sociodemographic and professional characterization of the nurses who agreed to participate in this stage of the study. The nurses had an average age of 38.38 ± 3.86 years and were mostly female (84.6%). Regarding the professional category, only 23.1% (*n* = 3) held the title of nurse specialist (Table 5).

When asked about their training in pain management, 76.9% reported having no training in this area before the training program made in this study. However, all respondents (*n* = 13) considered it important to receive training in pain assessment and pain management.

### 3.4. Assessment of Nurses’ Satisfaction with Strategies Implemented

The nurses’ satisfaction with the implemented strategies is described in Table 6. The average level of satisfaction was 7.92 ± 1.32 (on a scale from 0 to 10), with no assessments below 5.

The nurses considered the pain assessment intervention to be very important (61.5%), and 100% of the nurses believed it is important to have a pain assessment protocol during HD treatments.

Regarding the suitability of the implemented protocol in the clinical context, 69.2% of the nurses considered it suitable. Additionally, 92.3% agreed that the protocol was easy to apply, and 92.3% rated the protocol as effective for assessing pain during HD treatment.

When asked if “Patients’ pain management is more effective after the implementation of the protocol?” and “Was the protocol able to give visibility to pain assessment during HD treatment?” all the professionals (100%) answered “yes.”

Furthermore, 92.3% of the nurses reported that their approach to patients with pain during HD treatment had improved after the implementation of the protocol. All respondents (100%) said that the protocol contributed to improving care for patients with CKD undergoing HD treatment.

The second questionnaire also included two open-ended questions.

The first question collected suggestions to improve the pain approach during HD treatment. Only five of the thirteen nurses replied. The answers were categorized into two thematic areas: training (*n* = 3) and non-pharmacological pain management (*n* = 2). The respondents suggested that pain training should be provided to the entire multidisciplinary team, not just the nursing team (Q3; Q12 and Q13). They also recommended that non-pharmacological strategies should be implemented systematically and standardized by all team members (Q4 and Q8).

The second open-ended question related to suggestions for improving the implemented protocol. Four of the thirteen nurses made suggestions, all concerning recording software. They recommended improving the recording process by updating the current system to allow for the insertion of all information collected during pain assessments (Q1: “our record system should answer to our needs, and should allow us to insert pain assessments”; Q8: “the software needs to be update”).

## 4. Discussion

The quality improvement project revealed that the average age of patients studied was 71 ± 12.6 years, which is higher than the average age of HD treatment patients reported in the 2022 Annual Report of the Portuguese Society of Nephrology, which was 68.38 years [28]. This difference can be explained by the older demographic context verified in Centre of Portugal. An aging population and increased life expectancy are significant risk factors for developing CKD and are associated with a higher prevalence of diabetes and hypertension among patients [29]. The sample mainly consisted of male patients (64%), which corroborates the data presented by the Portuguese Society of Nephrology, indicating that 60% of patients undergoing HD treatment are male [30].

Regarding the presence of pain, 64% of patients reported experiencing daily pain, and 48% reported intradialytic pain. These results are corroborated by a study conducted by Sousa et al. [31], which identified a prevalence of chronic pain at 56.6% and intradialytic pain at 30.1%. However, another study showed higher levels of acute pain in CKD patients, with a prevalence of acute pain at 60% and chronic pain at 48% [9]. Ineffectively managed acute pain tends to persist and develop chronic characteristics [14].

Regarding the location of pain in the pre- and post-treatment period, the lower limbs (20%), lumbar region (20%), and bones and muscles (18.7%) were the most frequently mentioned by patients. These results were also identified in the study by Sousa et al. [31]. The predominance of these locations is mainly related to the progression of CKD and the presence of renal bone disease [17].

The study identified a pain intensity value of 5.46 (on a scale of 0–10) when assessed using the NRPS. When using the Qualitative Pain Scale, the most frequently reported pain intensity was moderate (n = 23). These data align with the results found in the study by Kusztal et al. [32], where 57% of patients reported moderate pain, with an average intensity of 5.01 ± 1.3, as assessed using the NRPS. However, in the study by Santos et al. [17], the prevalent pain intensity was mild (38.5%) when the Qualitative Pain Scale was used, while the pain intensity was 6.11 ± 0.42 when NRPS was used.

Regarding the characteristics of the pain, this study differs from previous ones, with “crushing” (32%) being the most frequently identified, in contrast to “stabbing pain,” which was the most common in other studies [20]. This difference can be explained by the difficulty patients have in characterizing their pain. Pain is a subjective experience involving a complex interaction of physiological, psychosocial, cultural, and environmental influences [33]. Various factors interfere with its characterization, such as beliefs, cultural and spiritual values, previous pain experiences, personality, age, the surrounding environment, and the patient’s interpretation of their pain in relation to the current situation [34,35].

Pain is associated with a substantial reduction in the ability to carry out ADLs [36]. When asked about limitations on ADLs, responses were evenly split (32% “yes”; 32% “no”). These results differ from those presented by Santos et al. [17], who found a high level of pain interference in ADLs. This discrepancy can be attributed to differences in the sample between the two studies, particularly in age; in the cited study, the average age of the patients was 55 years old [17].

It should also be noted that these differences may be related to methodological variations, such as differences in sample size, assessment instruments, and inclusion criteria. Patients who reported that pain interfered with their ADLs identified mobility limitations as the most significant issue. These findings are consistent with other studies showing that pain interferes with patients’ ADLs, including their ability to walk and the quality of their sleep [37].

Intradialytic pain was predominantly localized in the lower limbs (18.7%) and the lower back area (18.7%). Studies have shown higher percentages of pain in the lower limbs: around 60% in the study by Santos et al. [17], 42.3% in the study by Sousa et al. [31], and 47% in the study by Pozo et al. [38]. 

These results suggest that is very important to implement a systematic and frequent pharmacological and non-pharmacological strategy to relieve pain during dialysis treatment. These strategies may include changing positions, using cushions to reduce pressure zones, and other similar measures [17]. Regarding the type of pain, 20% of patients reported feeling pain with the characteristics of a “tingling”, followed by “cramping” (12%), which can be explained by the rapid removal of body fluid during HD treatment [8]. To reduce the occurrence of cramps during treatment, it is necessary for nurses to assess the patient at the beginning and throughout the treatment and adapt it to the patient’s needs [39].

Of the patients who reported feeling pain during HD treatment, 37.3% felt that it worsened throughout the session. Kusztal et al. [32] also reported that 28% of patients characterized their pain as continuous and worsening during HD treatment. Sousa et al. [31] stated that the immobility required to carry out HD treatment aggravates the experience of musculoskeletal (bones and muscles) pain.

Managing patients’ pain during HD treatments requires a multi-professional approach, incorporating both pharmacological and non-pharmacological measures for effective pain management. The results show that 36% of patients reported taking analgesic therapy regularly to relieve pain at home, and 14.7% reported taking analgesics during HD treatment. Sousa et al. [31] also found that 58.2% of patients used analgesic medication regularly to manage chronic pain. Fleishman et al. [40] added that 66.1% of patients commonly administered analgesic drugs, while only 24.5% chose to implement non-pharmacological measures.

The choice of the most appropriate analgesic for each patient should consider the cause, nature, and intensity of the pain [41]. However, the results show that the most used analgesic therapy for pain relief was paracetamol, which is corroborated by the study by Marzouq et al. [42], where 56.3% of patients used paracetamol. These results align with the guidelines issued by the National Kidney Foundation, which recommend paracetamol for the treatment of mild and moderate pain in patients with CKD [3]. The use of non-steroidal anti-inflammatory drugs (NSAIDs) is contraindicated due to the risk of nephrotoxicity [43]. Recent studies have demonstrated the efficacy of opioid therapy in relieving severe pain in patients with CKD, but its use must be individualized to achieve optimal pain relief [17,44]. 

The WHO recommends the use of the “pain ladder tool” to support professionals in making the best clinical decisions. This tool categorizes medication and interventions based on pain intensity for efficient pain management. Although initially developed for cancer patients, it can be adapted for use in CKD patients by adjusting prescriptions to avoid adverse effects [45,46].

As mentioned above, the use of pharmacological measures in patients with CKD should consider the pharmacokinetics of the drug and possible drug interactions to avoid side effects [9]. In this context, Coluzzi [41] states that for this group of patients, where analgesic therapy is not without risks, the use of non-pharmacological pain relief measures should be prioritized.

Pain management using non-pharmacological measures has been widely used in CKD patients, with their effectiveness proven in several studies. For example, muscle relaxation techniques during HD treatments have been shown to effectively reduce and relieve pain [47,48]. The application of warm or cold compresses has also proven effective in reducing pain associated with cramps and fatigue in patients undergoing HD treatment [49]. Dinis and Sousa [50] suggest using an anti-stress ball to relieve pain during the cannulation of vascular access.

The appropriate approach to achieving effective pain relief in patients with CKD faces several barriers, including a lack of awareness of the problem, insufficient education of health professionals, and fear of potential side effects of analgesic therapy, among others [41].

When asked about referral and follow-up in pain consultations, 45.3% of patients said they had never attended a medical follow-up to manage their pain. This highlights the need for multidisciplinary follow-up in the approach to patients with CKD. Coluzzi [41] emphasizes that the high incidence of chronic pain in CKD patients requires nephrologists to adopt a differentiated approach and make timely referrals to a pain treatment unit.

After implementing the interventions based on the results discussed above, the nurses who deliver care to patients undergoing HD treatment were consulted. The nurses had an average age of 38.38 ± 3.86 years, ranging from 35 to 49 years, and an average of 15.54 ± 4.17 years of professional experience, indicating significant professional expertise. These results align with data provided by the Portuguese Nurses Regulator [51], which revealed that in Portugal, most nurses are between 36 and 40 years old and are predominantly female (84.6%). This is also consistent with data from the National Institute of Statistics in 2022 [52].

One of the results that requires attention is the number of nurses with training in pain assessment and management; only 23.1% of the sample reported having training in this area before this study. This low percentage necessitates intervention and concern, as pain should be approached as the fifth vital sign. However, the nurses demonstrated self-awareness, with 100% agreeing on the importance of attending training in this area. Sutherland [53] also emphasizes the need for nurses to develop skills in pain management, as this is essential for preserving the dignity and autonomy of patients and significantly improving their QoL.

In the study, 61.5% of the nurses considered it very important to assess pain during HD treatments, and all of them believed that having a pain assessment protocol is important. They agreed that the protocol allowed for the standardization of nursing care.

In this context, the Order of Portuguese Nurses has recommended that clinical guidelines for pain assessment and control be established at the organizational level, along with the implementation of documentation systems to promote a uniform approach to pain assessment and management [21]. Additionally, the Directorate-General for Health, in its National Plan for the Prevention and Control of Pain, has recommended the implementation of technical guidelines for the assessment and management of pain [27].

The importance of standardization and evidence-based recommendations likely explains the nursing team’s high level of satisfaction with the implemented protocol (7.92 ± 1.32). Regarding suggestions for improvement, the nursing team emphasized the need to enhance the nursing records process by creating a pain assessment item in the application used at an institutional level. The Order of Portuguese Nurses [54] also highlights that the use of computer applications for nursing records allows nursing practice to be documented, enables the monitoring of care quality and continuity, and provides greater visibility to nursing activities [53]. Bailas [55] adds that the use of information systems in nursing practice allows for quicker access to patient-related information and a more organized approach, promoting efficiency, increased productivity, and effective care.

The nurses also emphasized the need to focus on training multidisciplinary teams in pain management to improve the non-pharmacological approach to patients experiencing pain during HD treatments. The need for training in pain management is highlighted by Moyo and Madzimbamuto [56], who argue that the lack of knowledge among health professionals regarding pain management has serious consequences for patients.

A limitation of this study is the difficulty in mobilizing the entire nursing team to understand the importance of this issue, as well as the difficulty patients had in characterizing their pain, which could potentially bias the data collected.

For future studies, it would be pertinent to determine if there has been an effective change by the multi-professional team in the approach to pain management and assessment, evidenced by a reduction in the percentage of patients experiencing pain and an increase in patients’ quality of life. It is suggested that observational studies be conducted with these objectives, involving patients, nurses, and other members of the healthcare team.

## 5. Conclusions

Pain in CKD is a significant concern for health professionals, making detailed pain assessment essential. One of the objectives of this study was to assess pain characterization in CKD patients before, during, and after HD treatment. Given that pain is a subjective issue, it presents considerable difficulties and challenges for health professionals in achieving effective pain management. The study highlights the high prevalence of significant pain during HD treatments and the lack of pain monitoring by specialized pain management teams.

This reality underscores the necessity for a shift in the approach to CKD patients undergoing HD treatment by nurses and the multi-professional team, focusing on the patient as an individual with specific needs and respecting them accordingly.

Continuous quality improvement studies aimed at enhancing pain management in CKD are crucial and should be promoted by health managers. Effective chronic pain management not only leads to personal improvements and cost reduction but also enhances the satisfaction of health professionals involved in caring for patients with pain before, during, and after HD treatment.

The development of quality improvement projects aimed at better pain management in CKD is important and should be encouraged by health managers. It is recognized that effective chronic pain management, in addition to the personal improvements and benefits associated with cost reduction, also enhances the satisfaction of health professionals involved in caring for patients with pain before, during, and after HD treatment.

## Figures and Tables

**Table 5:** Professional Characterization of the Sample of Nurses.

		M	SD	Max	Min
Age (years)		38.38	3.86	49	35
Nursing experience (years)		15.54	4.18	26	10
		*n*	%		
Gender	Female	11	84.60		
	Male	2	15.40		
Professional category	General Nurse	10	76.90		
	Nurse Specialist	3	23.10		
Did you have some train in pain assessment/ management before this participation?	No	10	76.90		
	Yes	3	23.10		
Do you believe it is important to receive training in pain management?	Yes	13	100.00		

M—mean; SD—standard deviation; *n*—sample size; %—percentage; Min—minimum; Max—maximum.

**Table 6:** Assessment of Nurse Satisfaction.

		M	SD	Max	Min
How is your satisfaction level from (0–10), with the protocol implemented?		7.92	1.32	10	5
		*n*	%		
Do you believe it is important to assess pain during the HD treatment?	Important	5	38.50		
	Very Important	8	61.50		
Do you agree it is important to have a pain assessment protocol during the HD treatment?	Yes	13	100.00		
How do you rate the suitability of the protocol implemented for the clinical context?	Not Suitable	2	15.40		
	Suitable	9	69.20		
	Very Suitable	2	15.40		
In your opinion, is patients’ pain management more effective after protocol implementation?	Yes	13	100.00		
Do you think your approach to patients with pain has improved since the protocol implementation?	Yes	12	92.30		
	No	1	7.70		
Was the protocol able to give visibility to pain assessment during the HD treatment?	Yes	13	100.00		
Did you find the protocol easy to apply during the HD treatment?	Yes	12	92.30		
	No	1	7.70		
Do you agree that the protocol is effective for assessing pain during the HD treatment?	Yes	12	92.30		
	No	1	7.70		
Do you agree that the protocol has been properly implemented?	Yes	12	92.30		
	No	1	7.70		
Do you agree that adequate training/support has been given during the protocol implementation?	Yes	11	84,60		
	No	2	15.40		
Do you believe that the protocol has contributed to improving patient care?	Yes	13	100.00		

M—mean; SD—standard deviation; *n*—sample size; %—percentage; Max—maximum; Min—minimum.

## Data Availability

The data presented in this study are available on request from the corresponding author. The survey and pain protocol designed is also available through requesting.

## Public Involvement Statement

The participants engaged by responding to a survey regarding their perceptions about pain protocol implementation (nurses) and about pain characterization (patients in HD treatment). No software with artificial intelligence was used in any part of the study.

## Guidelines and Standards Statement

This manuscript adheres to the Standards for Quality Improvement Reporting Excellence (SQUIRE 2.0) guidelines for reporting quality improvement projects. The checklist provided by SQUIRE 2.0 (available at https://www.equator-network.org/reporting-guidelines/squire/ on 29 March 2024) has been consulted during the preparation of this manuscript to ensure comprehensive and transparent reporting of our study. The checklist is available in the Appendix A.

## Appendix A

**Table A1:** SQUIRE 2.0—Revised Standards for Quality Improvement Reporting Excellence.

Text Section and Item Name	Section or Item Description	Page No
Title and Abstract		
Title	Indicate that the manuscript concerns an initiative to improve healthcare (broadly defined to include the quality, safety, effectiveness, patient-centeredness, timeliness, cost, efficiency, and equity of healthcare)	1
2. Abstract	a. Provide adequate information to aid in searching and indexing b. Summarize all key information from various sections of the text using the abstract format of the intended publication or a structured summary such as: background, local problem, methods, interventions, results, conclusions	1
Introduction	Why did you start?	
3. Problem Description	Nature and significance of the local problem	1–3
4. Available knowledge	Summary of what is currently known about the problem, including relevant previous studies	1–3
5. Rationale	Informal or formal frameworks, models, concepts, and/or theories used to explain the problem, any reasons or assumptions that were used to develop the intervention(s), and reasons why the intervention(s) was expected to work	2
6. Specific aims	Purpose of the project and of this report	4
Methods	What did you do?	
7. Context	Contextual elements considered important at the outset of introducing the intervention(s)	3
8. Intervention	a. Description of the intervention(s) in sufficient detail that others could reproduce it b. Specifics of the team involved in the work	4
9. Study of the Intervention (s)	a. Approach chosen for assessing the impact of the intervention(s) b. Approach used to establish whether the observed outcomes were due to the intervention(s)	4
10. Measures	a. Measures chosen for studying processes and outcomes of the intervention(s), including rationale for choosing them, their operational definitions, and their validity and reliability b. Description of the approach to the ongoing assessment of contextual elements that contributed to the success, failure, efficiency, and cost c. Methods employed for assessing completeness and accuracy of data	4
11. Analysis	a. Qualitative and quantitative methods used to draw inferences from the data b. Methods for understanding variation within the data, including the effects of time as a variable	5
12. Ethical Considerations	Ethical aspects of implementing and studying the intervention(s) and how they were addressed, including, but not limited to, formal ethics review and potential conflict(s) of interest	6
Results	What did you find?	
13. Results	a. Initial steps of the intervention(s) and their evolution over time (e.g., time-line diagram, flow chart, or table), including modifications made to the intervention during the project b. Details of the process measures and outcome c. Contextual elements that interacted with the intervention(s) d. Observed associations between outcomes, interventions, and relevant contextual elements e. Unintended consequences such as unexpected benefits, problems, failures, or costs associated with the intervention(s). f. Details about missing data	6–12
Discussion	What does it mean?	
14. Interpretation	a. Nature of the association between the intervention(s) and the outcomes b. Comparison of results with findings from other publications c. Impact of the project on people and systems d. Reasons for any differences between observed and anticipated outcomes, including the influence of context e. Costs and strategic trade-offs, including opportunity costs	12–15
15. Limitations	a. Limits to the generalizability of the work b. Factors that might have limited internal validity such as confounding, bias, or imprecision in the design, methods, measurement, or analysis. c. Efforts made to minimize and adjust for limitations	15
16. Conclusions	a. Usefulness of the work b. Sustainability c. Potential for spread to other contexts d. Implications for practice and further study in the field e. Suggested next steps	15–16
